# Time Distribution of the Onset of Chest Pain in Subjects with Acute ST-Elevation Myocardial Infarction: An Eight-Year, Single-Center Study in China

**DOI:** 10.1371/journal.pone.0032478

**Published:** 2012-03-12

**Authors:** En-Zhi Jia, Zhen-Xia Xu, Hong-Zhou Cai, Chang–Yan Guo, Li Li, Tie-Bing Zhu, Lian-Sheng Wang, Ke-Jiang Cao, Wen-Zhu Ma, Zhi-Jian Yang

**Affiliations:** Department of Cardiovascular Medicine, First Affiliated Hospital of Nanjing Medical University, Nanjing, Jiangsu Province, China; Sapienza University of Rome, Italy

## Abstract

**Objective:**

The objective of this study was to explore the time distribution patterns of the onset of chest pain in subjects with acute ST-elevation myocardial infarction in a Chinese population.

**Methods:**

A total of 1467 patients with acute ST-elevation myocardial infarction were enrolled from 2003 to 2010. The hourly, daily, monthly, seasonal and day-of-week fluctuations in the prevalence of acute ST-elevation myocardial infarction were analyzed.

**Results:**

A peak was found between the morning hours of 07:31 and 08:30. A second peak was observed between 14:31 and 15:30, and a third peak was found between 23:31 and 00:30 (*p*<0.001). The monthly maximum was recorded in November and the minimum was in April (*p*<0.001). The number of daily cases was greatest in autumn and lowest in the spring (*p* = 0.001). Day-of-the-week variations of ST-elevation acute myocardial infarction were not found, except in patients more than 75-years-old.

**Conclusions:**

Periodic variations in the frequency of ST-elevation acute myocardial infarction in Chinese patients showed significant differences with regard to diurnal, monthly and seasonal patterns. The exact mechanisms underlying these circadian variations require further study.

## Introduction

Various studies have reported circadian variation in patients with acute myocardial infarction (AMI) [1]–[5]. A meta-analysis of studies on circadian variation in myocardial infarction and sudden cardiac death demonstrated that the impact is significant, because about one out of every 11 AMIs and one out of every 15 sudden cardiac deaths occur in the morning [6]. Population-based analyses of 24061 consecutive cases revealed that there were marked variations in the occurrence of sudden death, with peaks observed during the morning, on Mondays and during the winter months [7]. Recently, a retrospective database study discovered that the number of hours of daylight and the time of sunrise may be associated with heart attack risk [8]. In a prospective, observational study using a multicenter online registry of 4573 patients diagnosed with AMI in Korea showed that the highest incidence of AMI occurred between 08:00 (24-hour clock) and noon, with the number of cases being highest in the winter and lowest in the autumn [9]. Differences in the circadian variation of AMI in many regions of the world and in different ethnic groups have been reported [10]. However, limited information is available with regard to circadian variation of patients with ST-elevation myocardial infarction (STEMI) in China.

Accordingly, we prospectively explored the time distribution of the onset of chest pain in subjects with acute STEMI in a Chinese population.

## Materials and Methods

The study protocol was approved by the Ethics Committee of the First Affiliated Hospital of Nanjing Medical University (Nanjing, China). Written informed consent was obtained from each patient.

### Study subjects

This study was a prospective, observational single-center registry evaluation that investigated circadian variation in patients with STEMI in China. Consecutive adult patients with acute STEMI who presented at the First Affiliated Hospital of Nanjing Medical University from 1 January 2003 to 31 December 2010 were included in the study. The diagnosis of acute STEMI was defined as ≥30 min of continuous chest pain, ST-segment elevation >2.0 mm on ≥2 contiguous electrocardiographic leads, and more than a two-fold elevation in the creatine kinase-MB fraction (CKMB) level [11]. Exclusion criteria were cardiac shock, severe liver and/or renal dysfunction, history of allergic response to drugs, and severe hypovolemia. The rationale for these exclusion criteria was that the subjects with the above complications could not report the time of onset of acute STEMI accurately. The time of onset of acute STEMI was determined by the self-reported time of onset of chest pain. In total, 1467 subjects were included in this study. A total of 83 subjects were excluded from the study according to the above exclusion criteria. There was no significant difference in circadian monthly distribution of the excluded subjects (*vs.* included subjects, *p* = 0.087).

### Data analyses

Each day was divided into hours according to the 24-hour clock. Patients were grouped according to the time of symptom onset. In addition, seasons were defined according to geographic information: spring was considered to be from 1 March to 31 May; summer from 1 June to 31 August; autumn from 1 September to 30 November; and winter from 1 December to 28 February. We analyzed baseline demographic characteristics, initial presentation, initial vital signs, results of laboratory tests, and discharge medications of the patients. The following demographic and clinical characteristics were recorded: age, sex, body mass index (BMI), and cardiovascular risk factors (hypertension, diabetes mellitus (DM), hyperlipidemia, and current smoking).

### Statistical analyses

Data were analyzed using the Statistics Package for Social Sciences (v16.0; SPSS Inc., Chicago, IL, USA). The Chi-squared goodness-of-fit test was conducted to measure the uniformity of the distribution of patients among the time periods.

The Chi-squared test was also used to determine if there was an even circadian distribution of cases during each hour of the day, and if each day differed with respect to circadian variation. The weekly distribution of cases was analyzed, comparing the number of subjects for each weekday. Finally, the monthly and seasonal occurrence of acute STEMI was examined. Because of the different number of days in each month (28 to 31 days), the number of events in each month was adjusted by dividing the number of cases by the number of days in the related month and multiplying by 30; this was done only in the monthly and daily analyses.

To account for skewing, the data of age and vital signs were expressed as median and quartile ranges, and comparisons were performed by the Mann–Whitney U-test. Categorical variables were compared between the groups of patients by Chi-squared test. In order to describe the population qualitatively, rather than only quantitatively, the logistic regression analysis was employed to evaluate impact of baseline factors on time distribution in the major *vs.* minor frequency incidence periods. Differences were considered to be significant if the null hypothesis could be rejected with >95% confidence. All *p*-values were two-tailed.

## Results

### Clinical characteristics according to sex

The clinical characteristics of patients are shown in Table 1. A total of 1467 patients (1159 males and 308 females; median age: 65 years (range: 55–74 years)) were enrolled in the study. Of these, 837 patients had anterior-wall AMI, 581 patients had inferior-wall AMI, and 49 patients had lateral-wall AMI.

**Table 1:** Clinical characteristics of subjects according to sex.

*Variable*	*Overall, N = 1467*	*Male, N = 1159*	*Female, N = 308*	*Z or Chi-square test*	*p-value*
Age, years	65 (55–74)	63 (54–72)	70 (63–77)	−8.109	<0.001
Body temperature, °C	36.7 (36.5–36.9)	36.7 (36.5–36.9)	36.7 (36.5–36.9)	−0.352	0.725
Heart rate, beats/minute	75 (65–85)	75 (65–85)	76 (65–87)	−0.595	0.552
Systolic blood pressure, mmHg	120 (110–135)	120 (110–135)	126 (110–140)	−3.533	<0.001
Diastolic blood pressure, mmHg	75 (70–81)	75 (70–80)	75 (68–83)	−0.519	0.604
Respiratory rate, breaths/min	18 (18–20)	18 (18–20)	19 (18–20)	−3.562	<0.001
Anterior-wall AMI (%)	837	655 (56.5)	182 (59.1)	0.659	0.417
Inferior-wall AMI (%)	581	464 (40.0)	117 (38.0)	0.426	0.514
Lateral-wall AMI (%)	49	40 (3.5)	9 (2.9)	0.211	0.646
Hyptertension (%)	814	636 (54.9)	178 (57.8)	0.838	0.360
Diabetes mellitus (%)	348	256 (22.1)	92 (29.0)	8.144	0.004
Current smoker (%)	649	629 (54.3)	20 (6.5)	225.170	<0.001

Data are presented as average values (range) or percentage.

There were no significant differences between male and females in body temperature (°C), pulse rate (beats per minute), diastolic blood pressure (DBP; mmHg), location of AMI, or history of hypertension. In the female AMI group, age (*p*<0.001), systolic blood pressure (SBP; mmHg; *p*<0.001), respiratory rate (breaths per minute; *p*<0.001), and history of DM (*p* = 0.004) were significantly higher, whereas the number of current smokers (*p*<0.001) was significantly lower, than in the male AMI group.

### Hourly distribution of STEMI

**Figure 1:**
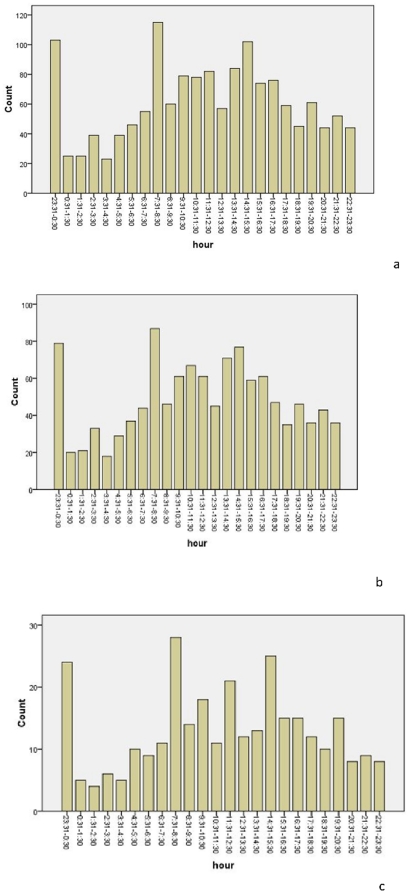
The hourly fluctuation for acute myocardial infarction in a) both sexes combined, b) males, and c) females.

Table 2 and Figure 1 show the distribution of STEMI, by hour, for both sexes combined. A significant circadian variation between males and females was found (*p*<0.001). The minimum prevalence occurred between 03:31 and 04:30. A peak was found in the morning hours between 07:31 and 08:30, and the prevalence remained high until noon. The prevalence decreased in the early afternoon, and began to rise again to reach a secondary peak between 14:31 and 15:30. A third peak was found between 23:31 and 00:30.

**Table 2:** Sex-specific prevalence of acute myocardial infarction by hour of the day.

*Hour*	*Overall*	*Statistical parameter*	*Male*	*Statistical parameter*	*Female*	*Statistical parameter*
23:31–0:30	103	*X* ^2^ = 237.85*p*<0.001	79	*X* ^2^ = 175.49*p*<0.001	24	*X* ^2^ = 74.753*pP*<0.001
00:31–01:30	25		20		5	
01:31–02:30	25		21		4	
02:31–03:30	39		33		6	
03:31–04:30	23		18		5	
04:31–05:30	39		29		10	
05:31–06:30	46		37		9	
06:31–07:30	55		44		11	
07:31–08:30	115		87		28	
08:31–09:30	60		46		14	
09:31–10:30	79		61		18	
10:31–11:30	78		67		11	
11:31–12:30	82		61		21	
12:31–13:30	57		45		12	
13:31–14:30	84		71		13	
14:31–15:30	102		77		25	
15:31–16:30	74		59		15	
16:31–17:30	76		61		15	
17:31–18:30	59		47		12	
18:31–19:30	45		35		10	
19:31–20:30	61		46		15	
20:31–21:30	44		36		8	
21:31–22:30	52		43		9	
22:31–23:30	44		36		8	

The numbers indicate the total count for each hour.

### Daily distribution of STEMI

**Figure 2:**
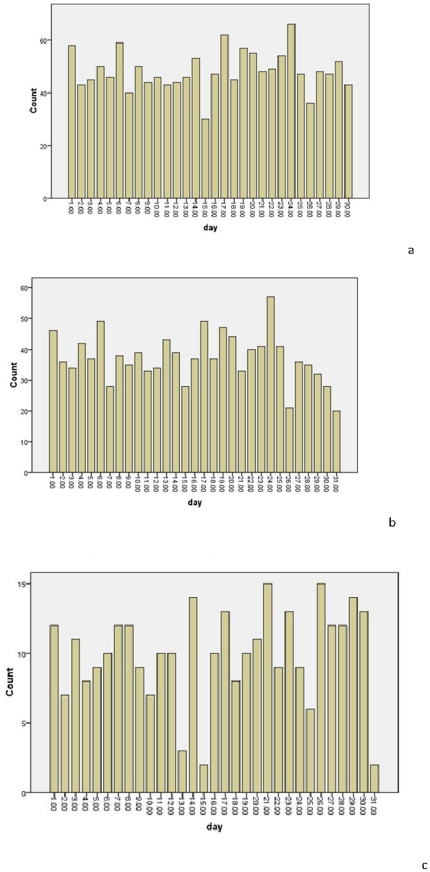
The day-of-the-week fluctuation for acute myocardial infarction in a) both sexes combined, b) males, and c) females.

The daily distribution of STEMI is shown in Table 3 and Figure 2. There was no definitive difference observed in the day of week of STEMI onset in the study sample (*p* = 0. 257).

**Table 3:** Sex-specific prevalence of acute myocardial infarction by day of the month.

*Day*	*Overall*	*Statistical parameter*	*Male*	*Statistical parameter*	*Female*	*Statistical parameter*
1	58	*X* ^2^ = 33.518*p* = 0.257	46	*X* ^2^ = 40.633*p* = 0.074	12	*X* ^2^ = 29.597*p* = 0.434
2	43		36		7	
3	45		34		11	
4	50		42		8	
5	46		37		9	
6	59		49		10	
7	40		28		12	
8	50		38		12	
9	44		35		9	
10	46		39		7	
11	43		33		10	
12	44		34		10	
13	46		43		3	
14	53		39		14	
15	30		28		2	
16	47		37		10	
17	62		49		13	
18	45		37		8	
19	57		47		10	
20	55		44		11	
21	48		33		15	
22	49		40		9	
23	54		41		13	
24	66		57		9	
25	47		41		6	
26	36		21		15	
27	48		36		12	
28	47		35		12	
29	52		37		14	
30	43		28		15	

The numbers indicate the total count for each day.

### Monthly distribution of STEMI

**Figure 3:**
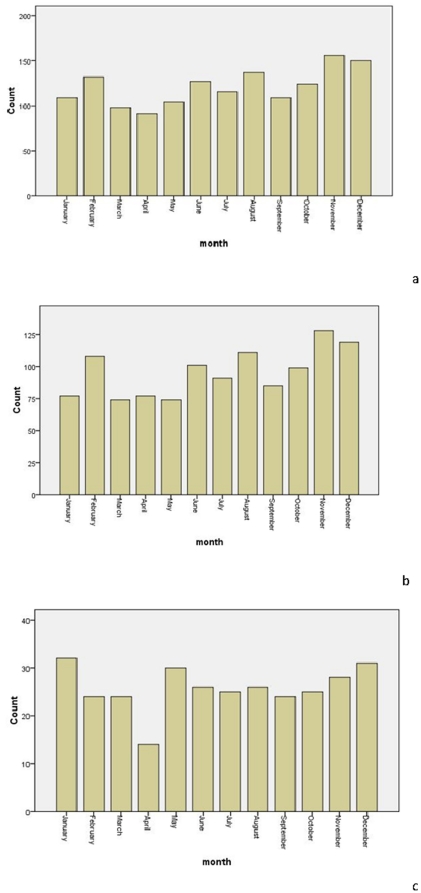
The monthly fluctuation for acute myocardial infarction in a) both sexes combined, b) males, and c) females.

Table 4 and Figure 3 show the monthly numbers of STEMI cases. For all patients, the monthly maximum was recorded in November and the minimum in April (*p*<0.001). For males, the monthly maximum was recorded in November and the minimum in March and May (*p*<0.001). However, a significant difference in circadian monthly distribution of STEMI was not found for the females (*p* = 0. 580).

**Table 4:** Sex-specific prevalence of acute myocardial infarction by month of the year.

*Month*	*Overall*	*Statistical parameter*	*Male*	*Statistical parameter*	*Female*	*Statistical parameter*
January	109	*X* ^2^ = 37.321*p*<0.001	77	*X* ^2^ = 39.720*p*<0.001	32	*X* ^2^ = 9.455 *p* = 0.580
February	132		108		24	
March	98		74		24	
April	91		77		14	
May	104		74		30	
June	127		101		26	
July	116		91		25	
August	137		111		26	
September	109		85		24	
October	124		99		25	
November	156		128		28	
December	150		119		31	

The numbers indicate the total count for each month.

### Seasonal distribution of STEMI

**Figure 4:**
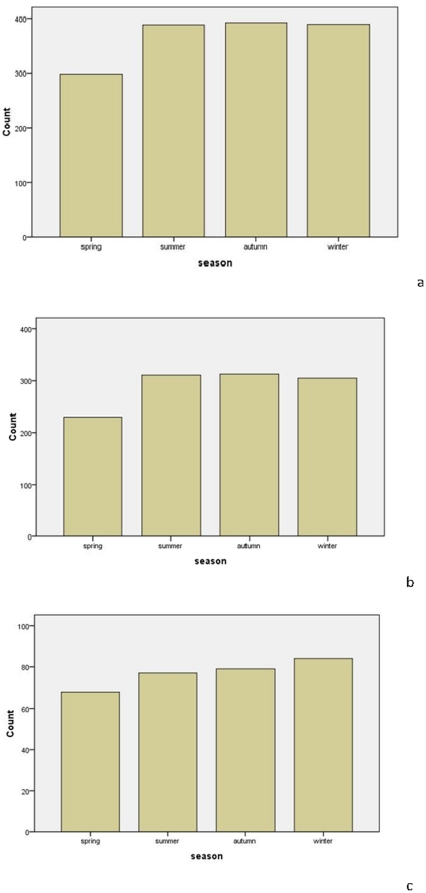
The seasonal fluctuation for acute myocardial infarction in a) both sexes combined, b) males, and c) females.

The sex-specific prevalence of STEMI by season is shown in Table 5 and Figure 4. In males, during the eight-year study period, the number of cases of daily STEMI onset was greatest in the autumn, then gradually decreased and reached the lowest value in the spring (*p* = 0.001). However, there were no significant differences in the number of cases of daily STEMI onset among seasons for the females (*p* = 0.628).

**Table 5:** Gender-specific prevalence of acute myocardial infarction per season.

*Season*	*Overall*	*Statistical parameter*	*Male*	*Statistical parameter*	*Female*	*Statistical parameter*
Spring	298	*X* ^2^ = 17.207 *p* = 0.001	230	*X* ^2^ = 16.548*p* = 0.001	68	*X* ^2^ = 1.740 *p* = 0.628
Summer	388		311		77	
Autumn	392		313		79	
Winter	389		305		84	

The numbers indicate the total count for each season.

### Day-of-the-week distribution of STEMI

**Figure 5:**
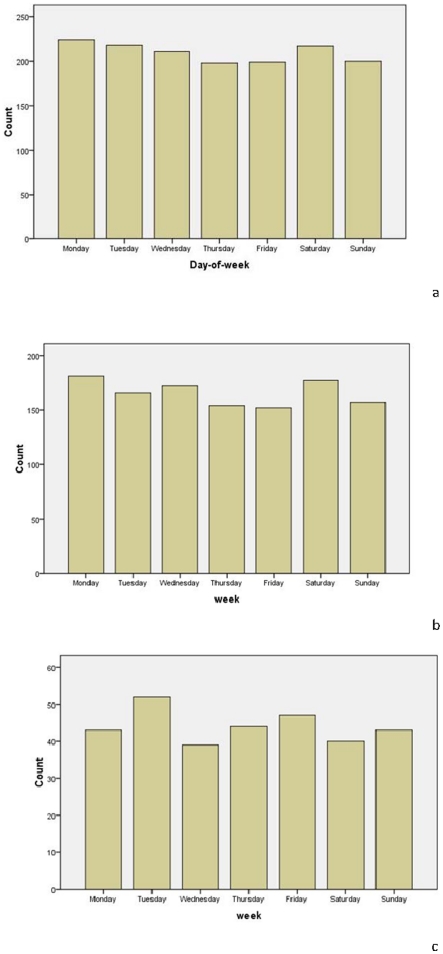
The day-of-weekly fluctuation for acute myocardial infarction in a) both sexes combined, b) males, and c) females.

**Figure 6:**
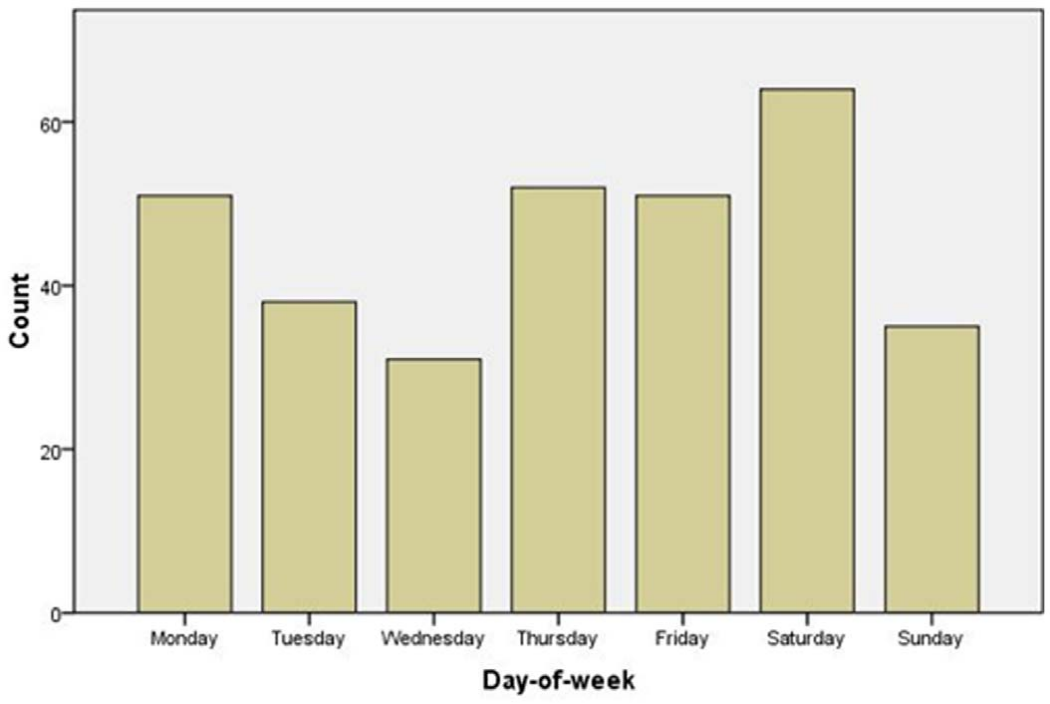
The day-of-the-week fluctuation for acute myocardial infarction in patients ≥75 years-old.

Table 6 and Figure 5 detail the day-of-week distributions of STEMI during the eight-year study period. There was no significant difference among the weekdays, regardless of sex (*p* = 0.781 for total, *p* = 0. 564 for males, *p* = 0.853 for females). However, in the sub-analysis for quartile of age, a significant difference was found among the day-of-the-week in the fourth group (more than 75-years-old); the highest number of AMI cases occurred on Saturday (*p* = 0.007; Table 7 and Figure 6).

**Table 6:** Sex-specific prevalence of acute myocardial infarction by day-of-the-week.

Day-of-the-week	*Overall*	*Statistical parameter*	*Male*	*Statistical parameter*	*Female*	*Statistical parameter*
Monday	224	*X* ^2^ = 3.215 *p* = 0.781	181	*X* ^2^ = 4.842 *p* = 0.564	43	*X* ^2^ = 2.636 *p* = 0.853
Tuesday	218		166		52	
Wednesday	211		172		39	
Thursday	198		154		44	
Friday	199		152		47	
Saturday	217		177		40	
Sunday	200		157		43	

The numbers indicate the total count for each day of the week.

**Table 7:** Gender-specific incidence of acute myocardial infarction per day-of-the-week, with sub-group analyses for quartiles of age.

*Day-of-the-week*	*Overall*	*Statistical parameter*	*Male*	*Statistical parameter*	*Female*	*Statistical parameter*
Age group: ≤55						
Monday	63	*X* ^2^ = 10.340 *p* = 0.111	56	*X* ^2^ = 9.170 *p* = 0.164	7	*X* ^2^ = 7.556 *p* = 0.273
Tuesday	60		53		7	
Wednesday	59		57		2	
Thursday	38		35		3	
Friday	42		38		4	
Saturday	52		48		4	
Sunday	57		48		9	
Age group: 56–65						
Monday	52	*X* ^2^ = 2.128 *p* = 0.908	46	*X* ^2^ = 2.227 *p* = 0.898	6	*X* ^2^ = 4.212 *p* = 0.648
Tuesday	51		40		11	
Wednesday	56		44		12	
Thursday	53		41		12	
Friday	51		42		9	
Saturday	49		43		6	
Sunday	62		52		10	
Age group: 66–74						
Monday	58	*X* ^2^ = 6.350 *p* = 0.385	47	*X* ^2^ = 5.858 *p* = 0.439	11	*X* ^2^ = 8.133 *p* = 0.228
Tuesday	69		48		21	
Wednesday	65		48		17	
Thursday	55		42		13	
Friday	55		35		20	
Saturday	52		43		9	
Sunday	46		32		14	
Age group: ≥75						
Monday	51	*X* ^2^ = 17.826 *p* = 0.007	32	*X* ^2^ = 10.760 *p* = 0.096	19	*X* ^2^ = 8.990 *p* = 0.174
Tuesday	38		25		13	
Wednesday	31		23		8	
Thursday	52		36		16	
Friday	51		37		14	
Saturday	64		43		21	
Sunday	35		25		10	

### Logistic regression analysis

Table 8 displays the results of logistic regression analysis to evaluate the impact of baseline characteristics on hourly, monthly and seasonal time distribution of STEMI. There was no significant impact of baseline factors on hourly, monthly or seasonal distribution of STEMI in major *vs.* minor frequency incidence periods, except for the body temperature on the seasonal distribution.

**Table 8:** The logistic regression analysis of baseline factors on time distribution in major *vs.* minor event incidence of STEMI.

*Baseline factors*	*Hourly distribution, 03:31–04:30 vs. 07:31–08:30*		*Monthly distribution, November vs. April*		*Seasonal distribution, Autumn vs. Spring*	
	*OR*(95% *CI*)	*p*-value	*OR*(95% *CI*)	*p*-value	*OR*(95% *CI*)	*p*-value
Age, years	0.774(0.475–1.261)	0.303	0.980(0.764–1.256)	0.871	0.956(0.825–1.107)	0.546
Sex	0.871(0.219–3.456)	0.844	1.099(0.488–2.476)	0.820	0.744(0.487–1.137)	0.172
Body temperature, °C	1.045(0.643–1.699)	0.859	0.920(0.723–1.171)	0.500	0.812(0.698–0.944)	0.007
Heart rate, beats/minute	0.763(0.490–1.189)	0.233	0.933(0.731–1.191)	0.578	0.929(0.806–1.070)	0.306
Systolic blood pressure, mmHg	1.265(0.599–2.669)	0.537	1.075(0.739–1.565)	0.704	1.165(0.949–1.431)	0.145
Diastolic blood pressure, mmHg	0.870(0.424–1.784)	0.870	1.153(0.808–1.646)	0.432	1.041(0.851–1.273)	0.696
Respiratory rate, breaths/min	1.734(0.595–5.053)	0.313	1.390(0.797–2.425)	0.246	0.904(0.658–1.242)	0.532
Anterior-wall AMI	0.000	0.999	0.416(0.044–3.927)	0.444	0.601(0.251–1.441)	0.254
Inferior-wall AMI	0.000	0.999	0.315(0.033–3.007)	0.316	0.607(0.249–1.475)	0.270
Hypertension	2.160(0.735–6.343)	0.161	1.137(0.651–1.985)	0.651	1.296(0.937–1.793)	0.117
Diabetes mellitus	0.913(0.318–2.617)	0.865	1.325(0.695–2.525)	0.393	0.922(0.633–1.342)	0.671
Current smoker	0.770(0.242–2.446)	0.657	0.917(0.500–1.680)	0.778	0.796(0.561–1.130)	0.202

## Discussion

This was the largest study to date analyzing periodic variation and STEMI in Chinese patients. The periodic variation in the frequency of STEMI in Chinese patients was significantly different with regard to diurnal, monthly and seasonal patterns. In addition, the monthly and seasonal variations showed a difference between males and females. We found no day-of-the-week variation of STEMI in Chinese patients, except for patients ≥75 years of age.

After Pell and d'Alonzo first identified an association between circadian variation and AMI [12], several studies reported similar patterns and revealed that the peak onset occurs in the morning [1]–[3]. In the present study, the periodic variation in the frequency of STEMI in Chinese patients showed similar diurnal patterns to patients from previous reports, in that the peak prevalence occurred in the early morning. However, we found three peaks of STEMI in Chinese patients; this finding was different to that seen in other reports [1]–[3], [6], [9]. In the present study, a morning peak occurred between 07:31 and 08:30, the second peak was between 14:31 and 15:30, and the third peak was between 23:31 and 00:30. While the exact mechanism underlying this circadian variation is not known, the endogenous circadian system may contribute to these daily fluctuations. One study suggested that the circadian system modulates numerous cardiovascular risk markers, both at rest as well as during exercise, with profiles that could potentially contribute to the day/night pattern seen for adverse cardiovascular events [13]. The switch from sleeping to activity may partially account for the daily fluctuations of AMI occurrence, but the exact mechanisms require further study.

We found that the prevalence of STEMI in Chinese patients was highest in November and lowest in April, when both sexes were included in the analysis. A previous study from Spain revealed the highest incidence of AMI to be in January and the lowest to be in August [14]. A German survey discovered the highest incidence of AMI to be in December and January and the lowest to be in July [15]. Using data from a Korean AMI registry, the monthly prevalence was found to be highest in January and lowest in October [16]. The results of the present Chinese-based study differ from those previous findings, and this difference could be attributed to geographical and climatic variations.

The concept that climate can effect health goes back to at least the time of Hippocrates (430 BC). He described the correlation between environmental conditions and pathogenesis of disease in his treatise “Of Airs, Waters and Places” [17]. In recent years, much attention has been given to the seasonal variation of AMI. The recent CLIMATE study in Athens, Greece, indicated that the seasonal variation in deaths by AMI was significant, with the average daily deaths due to AMI being 31.8% higher in winter than in summer (9.89 per day *vs.* 7.35 per day, *p*<0.001). The best predictor of daily deaths due to AMI was reported to be the average temperature of the previous seven days; with the relationship between daily AMI deaths and the seven-day average temperature being U-shaped [18]. Data from a Korean AMI registry showed that seasonal variations of AMI existed and were characterized by a winter peak and a summer trough (*p*<0.001). In addition, there were significant associations between hospital admissions and other meteorological parameters, including air temperature, relative humidity, and sunshine duration, even after adjusting for the effects of the day-of-week, season, and holidays (*p*<0.05) [16]. In the present study, the peak period of STEMI was the autumn, with the lowest prevalence of events in the spring. Due to meteorological parameters having a significant influence on the occurrence of AMI, meteorological variations may have contributed to the differences between the findings of the present study and previous reports.

There are a few reports noting that the peak of AMI occurrs on a Monday morning in working populations [19]. A potential mechanism to explain the highest number of cases of AMI occurring on Monday morning may be attributed to stress on the vascular and nervous systems triggered by psychological and physical factors on the first working day of the week [16]. There was, however, no significant difference in AMI prevalence among the day-of-the-week in the present study. Results from sub-analysis did reveal that the highest number of AMI cases in subjects more than 75 years-old occurred on Saturday. In these cases, the circadian variation was also observed. The mechanism underlying this phenomenon needs to be explored in a future study.

The present study has several limitations. First, the association of climatic seasonal variations with STEMI onset was not a focus of this study. A few studies have indicated that certain meteorological factors may be related to the onset of AMI [10], [16], [20], [21]. Therefore, the effect of climatic condition on the onset of STEMI should be explored in future studies. Second, the time distribution of the onset of chest pain in subjects with acute ST-elevation myocardial infarction in the present study was determined in an eight-year, single-center analysis, rather than by using a multi-center approach. Finally, the objective of this study was to explore the time distribution of the onset of chest pain in subjects with acute ST-elevation myocardial infarction. The activity level of the patients at the moment of the onset of the chest pain, such as whether they were sleeping and in activity, was not investigated. A multi-center prospective epidemiological study is warranted, to address the association of activity with the onset of STEMI.
